# Bubble-pen lithography: Fundamentals and applications: Nanoscience: Special Issue Dedicated to Professor Paul S. Weiss

**DOI:** 10.1002/agt2.189

**Published:** 2022-03-08

**Authors:** Pavana Siddhartha Kollipara, Ritvik Mahendra, Jingang Li, Yuebing Zheng

**Affiliations:** 1 Walker Department of Mechanical Engineering, The University of Texas at Austin, Austin, Texas, USA; 2 Department of Electrical and Computer Engineering, The University of Texas at Austin, Austin, Texas, USA; 3 Material Science and Engineering Program, Texas Materials Institute, The University of Texas at Austin, Austin, Texas, USA

**Keywords:** additive manufacturing, capillary flow, lithography, marangoni convection, microbubbles, sensing

## Abstract

Developing on-chip functional devices requires reliable fabrication methods with high resolution for miniaturization, desired components for enhanced performance, and high throughput for fast prototyping and mass production. Recently, laser-based bubble-pen lithography (BPL) has been developed to enable sub-micron linewidths, in situ synthesis of custom materials, and on-demand patterning for various functional components and devices. BPL exploits Marangoni convection induced by a laser-controlled microbubble to attract, accumulate, and immobilize particles, ions, and molecules onto different substrates. Recent years have witnessed tremendous progress in theory, engineering, and application of BPL, which motivated us to write this review. First, an overview of experimental demonstrations and theoretical understandings of BPL is presented. Next, we discuss the advantages of BPL and its diverse applications in quantum dot displays, biological and chemical sensing, clinical diagnosis, nanoalloy synthesis, and microrobotics. We conclude this review with our perspective on the challenges and future directions of BPL.

## 1 | INTRODUCTION

Recent years have witnessed an exponential growth in the development and implementation of functional miniaturized devices, such as point-of-care sensors,^[1–4]^ high-density transistor chips,^[5]^ planar batteries,^[6]^ augmented reality glasses,^[7,8]^ and micro-electro-mechanical systems.^[9]^ Conventional top-down lithographical techniques, such as photolithography,^[10–12]^ nanoimprinting lithography,^[13–15]^ electron beam lithography, and focused-ion beam lithography,^[16,17]^ have been developed to fabricate these devices with high fidelity. However, they generally suffer from expensive instruments, complex operations, and high materials wastage. In addition, the high-throughput fabrication of multi-component and three-dimensional (3D) structures is challenging.

To overcome these limitations, researchers proposed bottom-up methods as promising alternatives for the fabrication of functional devices.^[18]^ In comparison to conventional top-down techniques, bottom-up approaches provide additional flexibility in controlling the composition and configuration of functional structures.^[19]^ A typical bottom-up approach like printing involves two steps: (1) synthesis of functional materials as inks,^[20–22]^ and (2) printing of the inks into designed configurations on substrates for functional devices.^[23–26]^ Many techniques, such as hydrothermal synthesis,^[27]^ sol-gel synthesis,^[28,29]^ physical or chemical vapor deposition,^[30]^ and pyrolysis,^[31,32]^ have been developed to produce inks that are solutions with colloidal particles of precisely controllable sizes, shapes, compositions, and properties. These colloidal particles can serve as the building blocks for the construction of diverse functional structures where the properties can be tuned by both the discrete particles and the inter-particle interactions. Bottom-up fabrication starting from these building blocks is promising for producing multi-component and multi-functional structures with single-particle resolution and low materials wastage. Several bottom-up technologies have been developed, such as self-assembly^[33–35]^ and inkjet printing.^[36,37]^ However, self-assembly lacks precise control over the patterning geometries and is limited to thermodynamically stable configurations. Inkjet printing suffers from poor resolution, strict requirements on ink parameters, and the coffee ring effect.^[38]^ Laser-based fabrication techniques such as laser-induced hydrothermal synthesis^[39–42]^ and laser sintering^[43–45]^ offer unique advantages of high diffraction-limited resolutions and on-demand two-dimensional (2D) and 3D structure generation. However, laser-induced hydrothermal synthesis is limited by long laser exposure times and poor control of the 3D structure morphology. Laser-sintering provides a good prototyping alternative for electrical and pharmaceutical applications.^[46,47]^ Typically, laser sintering uses a femtosecond laser to fuse the particles to achieve the required 2D/3D patterns on diverse substrates. Although scalability is achieved through larger beam size and fast laser-scanning speeds, challenges such as the resolution limit due to particle size, high surface roughness and high porosity of the finished products, thermal shrinking and warping of the printed structures, and thermal requirements on materials limit the wide-applicability of laser sintering technology.^[48]^

An emerging technique, bubble-pen lithography (BPL), has been developed to overcome some of the limitations in the current bottom-up fabrication methods.^[49]^ In brief, BPL exploits laser heating of the substrate or solution to generate light-controlled microbubbles for the concentration and immobilization of ink materials (e.g., colloids, polymers, biomolecules, and ions) on the substrate.^[50–52]^ In addition, the concentrated ink materials at the bubble interfacial regions that act as local nanoreactors facilitate the in situ synthesis and structuring of unconventional functional materials.^[53]^ The bubble size and printing resolution in BPL can be controlled by the laser power and exposure time, which is desired for the patterning of multiscale structures. In this review, we highlight the current progress in fundamentals, technologies, and applications of BPL. Specifically, we discuss the fundamentals of microbubble formation from both experimental and theoretical aspects and illustrate the laser-bubble-patterning parametric relationship in detail. In addition, we present the implementation of BPL in diverse applications and provide a broad perspective on the challenges and prospects of BPL.

## 2 | WORKING PRINCIPLE OF BPL

**FIGURE 1:**
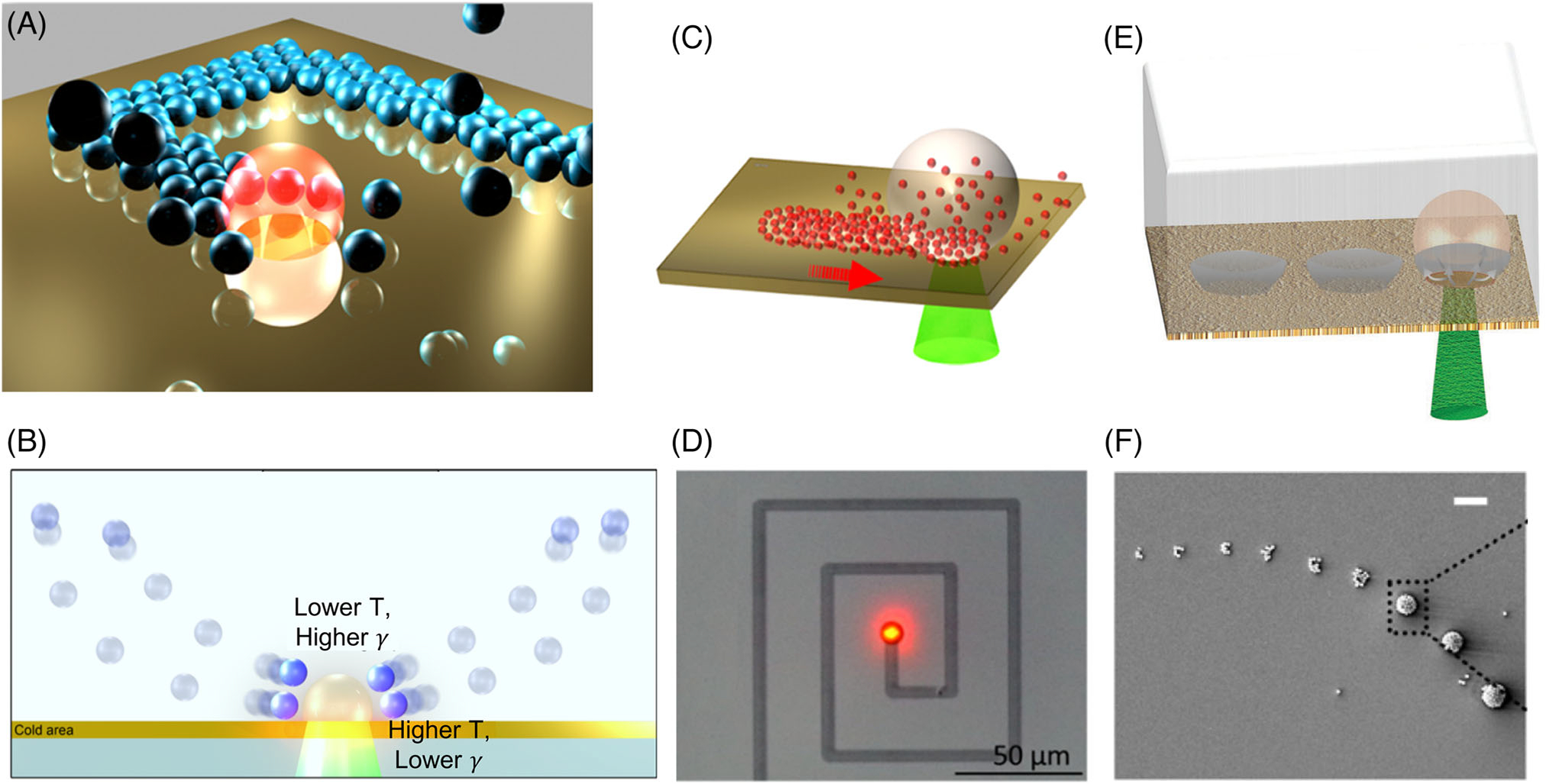
An overview of bubble-pen lithography (BPL): (A) Schematic of BPL. (B) Working principle of BPL. The surface tension (*γ*) decreases with increasing temperature (T). (C) Schematic of continuous printing via BPL. (D) Continuous printing of lines via BPL. (E) Schematic of discrete printing via BPL. (F) Discrete patterning of microparticle clusters via BPL. Scale bar: 5 *μ*m; (A, B, and F) Reproduced with permission: 2016, American Chemical Society.^[49]^ (C) Reproduced with permission: 2017, American Chemical Society.^[56]^ (D) Reproduced with permission: 2020, American Chemical Society.^[57]^ (E) Reproduced with permission: 2018, WILEY-VCH^[52]^

BPL is achieved through the control of optically generated microbubbles at the substrate-liquid interface (Figure 1A). Localized optical heating of the substrate/particle increases the temperature beyond the boiling point of the solvent, which leads to vaporization of the solvent and the formation of a bubble at the laser spot.^[54]^ The temperature difference across the microbubble corresponds to a steep temperature gradient of ~50–70 K/*μ*m for a typical 5 *μ*m bubble (Figure 1B).^[55]^ Since surface tension at the bubble interface is a function of temperature, the strong surface tension gradient results in Marangoni convection (also called thermocapillary convection), a swirling motion in the fluid above the surface.^[58]^ This convective flow leads to the attraction of inks in the solution within a range of 2–20 times the bubble size.^[59,60]^ Microbubble generation is nearly instantaneous, with a timescale in the order of microseconds. The increase of laser exposure time results in the continuous growth of the bubble until it is saturated. The steady bubble size is a function of solvent properties, laser power, and laser exposure duration.^[55]^

On-demand patterning on the substrate via BPL is achieved by the relative translation between the sample and the laser beam. As the heating spot shifts, the bubble continuously moves from one position to the other, which enables the continuous attraction of ink materials to the three-phase contact line for the immobilization and patterning on the substrate (Figure 1C).^[56]^ An example of experimental realization of continuous patterning via BPL is shown in Figure 1D.^[57]^ In addition to continuous printing, discrete printing is also possible by altering the “ON” and “OFF” state of the laser beam (Figure 1E).^[52]^ By using a high-speed shutter to block the laser beam, discrete patterning can be achieved. Figure 1F shows the discrete patterning of microparticle clusters on the substrate. Typically, continuous printing is extensively applied in conductometric sensing in biology and chemistry,^[4,61–63]^ while discrete patterning is commonly used for displays, interconnects, and multiplexed optical sensor arrays.^[64–66]^ Versatile patterning of diverse structures can be effectively achieved by altering the continuous and discrete patterning. The implementation of BPL for practical applications necessitates an in-depth understanding of the underlying physical mechanisms and a coordinated tuning of multiple parameters to control the printing resolution. In the following sub-sections, we present the experimental control and theoretical understanding of BPL, which have led to the enhanced capability of BPL.

### 2.1 | Experimental demonstration of BPL

**FIGURE 2:**
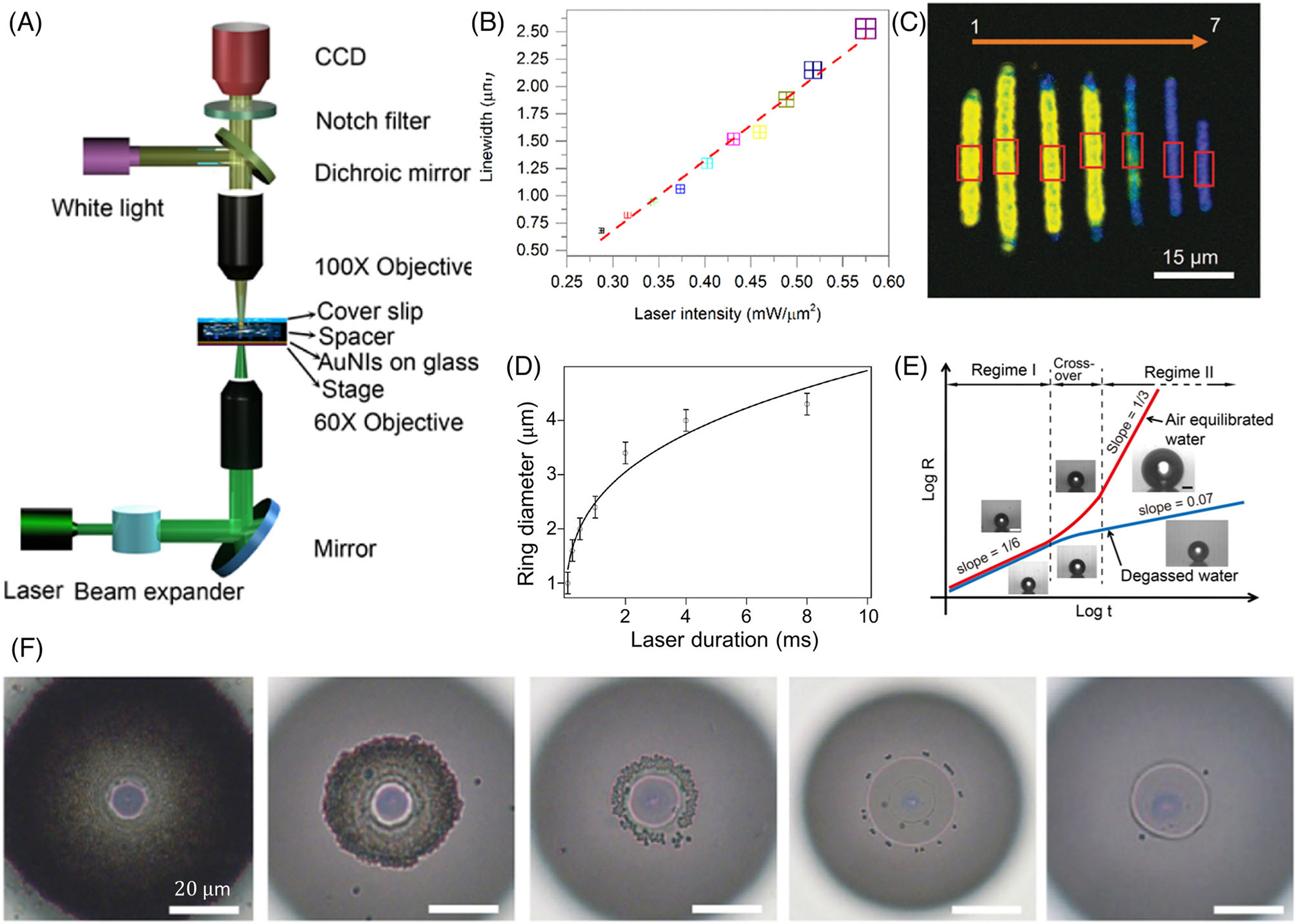
Experimental tunability of bubble-pen lithography (BPL): (A) A typical optical setup of BPL. Reproduced with permission: 2016, American Chemical Society.^[49]^ (B) Tunable printing linewidth by changing the laser intensity. Reproduced with permission: 2017, American Chemical Society.^[56]^ (C) Tunable linewidth and fluorescence by changing the laser scanning speed. Reproduced with permission: 2017, The Royal Society of Chemistry.^[79]^ (D) Tunable diameter of the printed rings by changing the laser exposure time. Reproduced with permission: 2013, American Chemical Society.^[78]^ (E) Tunable bubble size and bubble growth rate by changing the gaseous content of the solution. Reproduced with permission: 2017, Americal Chemical Society.^[81]^ (F) Tunable aggregation behavior by changing the particle concentration in the solvent from 4.55 × 10^8^ particles/ml (left panel) to 4.55 × 10^4^ particles/ml (right panel). The particle concentration decreased 10-fold for each panel from left to right. Reproduced with permission: 2019, American Chemical Society^[82]^

Figure 2A shows the typical optical setup to focus a laser beam onto the substrate for BPL. In this setup, optically generated microbubbles are created and moved through the digital control of the sample stage to fabricate designed patterns. Several factors in BPL, such as heating source, solvent, ink, and substrate, need to be considered to achieve the desired printing performance.

#### 2.1.1 | Heating source

BPL is initiated with optothermally induced microbubbles, which require an efficient light-to-heat conversion in the system for solvent boiling and bubble formation.^[67]^ This process can be achieved with the use of a combination of a light-absorbing substrate or a light-absorbing solution/dispersion with a relevant laser source. Plasmonic substrates are commonly used light-absorbing substrates in BPL.^[68,69]^ Plasmonic substrates can be obtained by the deposition of a thin layer (<10 nm) of metals on other surfaces, such as glass and polyethylene terephthalate (PET). A thermal annealing process is usually followed to convert the continuous thin film into discrete nanoislands to enhance the localized heating.^[70,71]^ Alternatively, plasmonic substrates can be prepared by the spin-coating or drop-casting of metal nanoparticle solution to deposit plasmonic nanoparticles onto the substrate. Both methods can lead to a relatively uniform distribution of metal nanoparticles and hotspots, and efficient light-to-heat conversion. Other light-absorbing substrates, such as indium-tin-oxide^[72–74]^ and silicon-based substrates,^[75,76]^ are also commonly used to convert light into heat. In addition, light-absorbing metal particles suspended in solvent can act as localized heat sources for plasmonic bubble generation.^[77]^ A high concentration of particles is usually required for efficient bubble-controlled patterning of such light-absorbing particles. In instances where the ink particles are not light-absorbing, a combination of ink particles and metal particles is utilized for effective bubble generation and continuous lithography.^[78]^

The other important parameters to control the optical heating include laser wavelength, laser power, and bubble exposure time. For example, plasmonic heating depends strongly on the laser wavelength relative to the plasmon resonance wavelength of the plasmonic substrate or dispersed plasmonic particle. Therefore, choosing the relevant laser wavelength is essential for a particular substrate or particle to generate bubbles at a lower laser power. As laser power increases, the bubble size and three-phase contact line expand, which increases the printing linewidth (Figure 2B).^[56]^ The printing linewidth is also dependent on the bubble exposure time, which can be controlled by the laser/stage scanning speed (continuous patterning) or the laser duration (discrete patterning). As the laser/stage scanning speed increases, the exposure time reduces, which decreases the accumulation time of the dispersed materials and reduces the patterning linewidth (Figure 2C).^[79]^ Similarly, Roy et al. demonstrated that the rings formed during the discrete patterning of composites had an increasing size along with the increasing laser duration (Figure 2D).^[78]^

#### 2.1.2 | Substrate

In addition to light absorption, the contact angle of bubbles on the substrate should also be optimized for the efficient immobilization of ink materials. For instance, Liu et al. demonstrated that the dynamics of generation and dissolution of plasmonic bubbles depend strongly on the wettability of the substrate.^[80]^ The light-to-heat conversion is weakened when the substrate is coated with hydrophobic polymers, increasing the threshold laser power for bubble generation. Microbubbles generated on hydrophobic substrates also grow slower than those on hydrophilic substrates. The size of the microbubble determines the temperature gradient across the bubble that alters the printing forces for the colloidal particles.

#### 2.1.3 | Dispersion

Bubble generation and its stability strongly depend on the viscosity, boiling point, solute concentration, and stabilizing surfactants of the ink dispersions, which should be controlled for the better performance of BPL.

##### Viscosity

As the solution viscosity increases, the Marangoni convection decreases due to the greater fluidic friction, which weakens the particle accumulation. Moreover, for a given size of the bubble, bubble manipulation becomes increasingly difficult due to an increase in the drag force on the bubble during laser/stage movement in higher viscosity solutions. Thus, low-viscosity solutions (<10 cP) such as water and alcohols are typically utilized for BPL.

##### Boiling point

The higher the boiling point of the dispersion, the higher the laser flux required to generate the bubble. If all other parameters are the same, it is easier for bubble formation in volatile liquids with lower boiling points than water.

##### Entrapped air in the solution

The bubble formation and growth dynamics strongly depend on the contents of the bubble – is it a vapor bubble or a gaseous bubble? Wang et al. presented a thorough understanding of bubble dynamics in the presence and absence of air in the dispersion (Figure 2E).^[81]^ During the instantaneous formation of a bubble (bubble nucleation), solvent around the heating spot is immediately vaporized to form a vapor bubble. Later, as laser heating continues, the air dispersed in the medium is attracted to the bubble, thus forming a mixture of air and solvent vapor inside the bubble. As the air continues to enter the bubble, the size of the bubble increases drastically, and an asymptotic size is achieved at longer time scales. If the dispersion is degassed, the effects of the entrapped air in the dispersion will be minimum, and the change rate of bubble size is much lower compared to that of bubble size in air-entrapped dispersions.^[81]^

##### Solute concentration

An increasing solute concentration can increase the particle accumulation at the three-phase contact line. Figure 2F shows the dependence of accumulation at varying particle concentrations (five orders of difference) at the same laser power and laser duration.^[82]^ In addition, if the heating sources are light-absorbing particles in the solution, a high concentration of solute particles is essential for efficient light-to-heat conversion.

##### Stabilizing surfactants

Surfactants are often utilized to synthesize and stabilize colloidal dispersions to prevent aggregate formation by inducing electrostatic repulsions among particles.^[83,84]^ In general, surfactants in the dispersion will be attracted to the bubble interface and reduce the surface tension magnitude and gradient, resulting in a decreased convection and range of particle attraction. In the absence of any counteracting chemical reactions or particle-surfactant interactions, the accumulation of particles thus reduces with the surfactant addition, resulting in smaller feature sizes of the printed structures. However, in some instances, additional surfactants can enhance the particle accumulation. Yamamoto et al. showed such a contrary response in the presence of a nonionic surfactant. Since the adsorption of nonionic surfactants is exothermic, the surfactant molecules are densely accumulated on the top portion of the microbubble (where the temperature is lower), thereby increasing the surface tension gradient near the three-phase contact line. This result caused an additional trapping force and led to a larger accumulation of the colloidal particles.^[82]^

We have briefly described the effects of these dispersion parameters on bubble formation and particle accumulation. Detailed theoretical analysis on the printing dynamics of BPL is still essential in promoting its practical applications, which will be discussed in the following sub-section.

### 2.2 | Theoretical analysis of BPL

**FIGURE 3:**
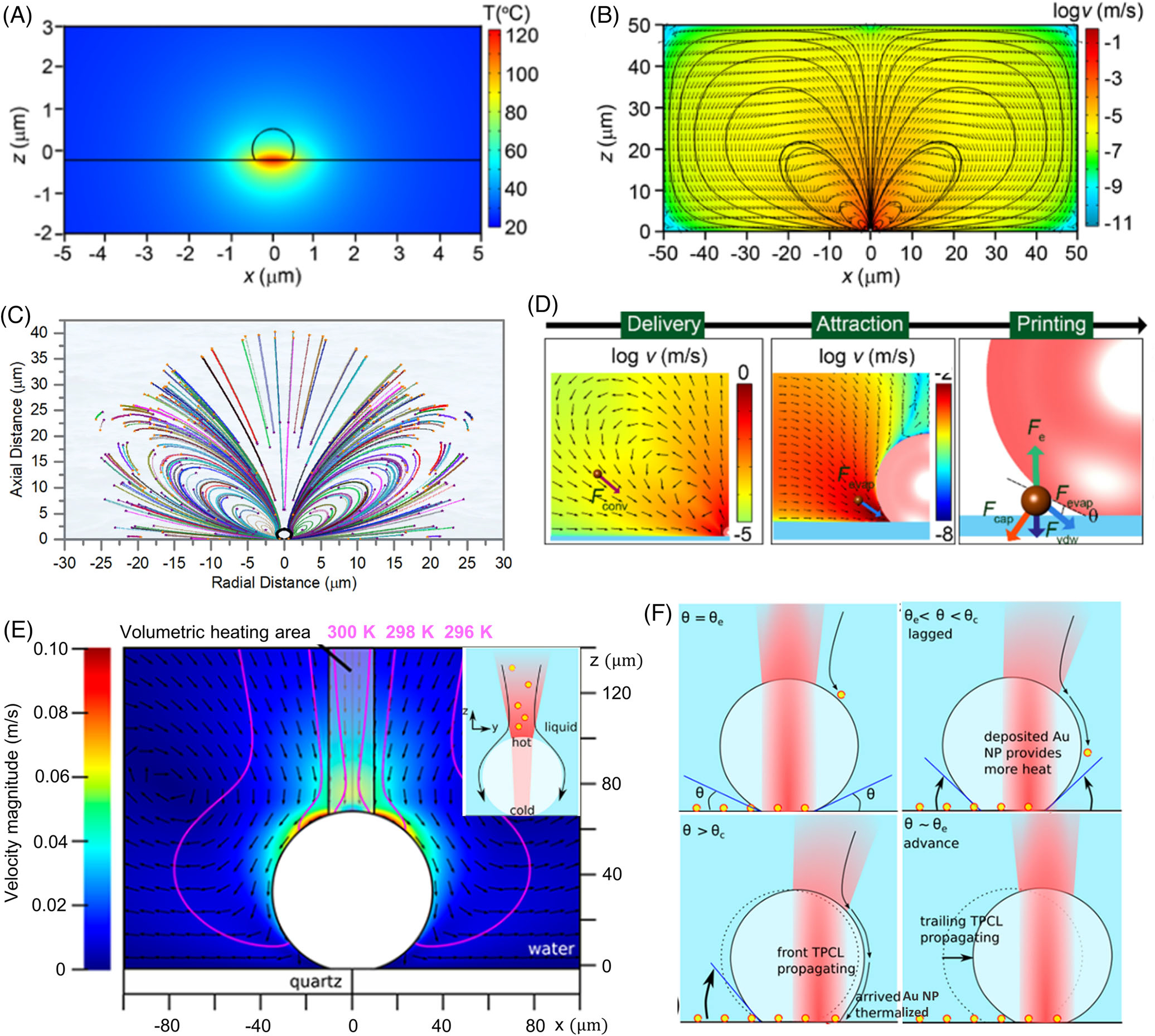
Fundamental understanding of bubble-pen lithography (BPL): (A) Temperature distribution around an optothermal microbubble generated on a light-absorbing substrate. (B) Resultant Marangoni convection due to the surface tension gradient at the bubble, spanning several tens of micrometers. (A and B) Reproduced with permission: 2016, American Chemical Society.^[49]^ (C) Random walk simulations demonstrating particle trajectories under the influence of Marangoni convection. Reproduced with permission: 2019, Elsevier Inc.^[53]^ (D) Force analysis on the particles: Drag force (*F*_conv_) attracts the particle toward the bubble because of Marangoni convection. In the vicinity of the bubble, the particle moves toward the three-phase contact line by the evaporation of the solution (*F*_evap_). Once the particle touches the bubble surface, capillary force (*F*_*cap*_), electrostatic force (*F*_*e*_), Van der Walls force (*F*_*vdw*_), and evaporative force (*F*_evap_) determine the printability of the particle on the substrate surface. Reproduced with permission: 2021, American Chemical Society.^[87]^ (E) For bubbles generated by light-absorbing particles, the direction of Marangoni flow on the bubble surface is opposite to that in the light-absorbing substrate. Inset: The heating source is the interaction of refracted laser beam and the light-absorbing particles above the substrate, which causes the local temperature to increase on the top of the bubble. (F) The particles are attracted toward the three-phase contact line due to the asymmetry in the refracted beam during laser motion. (E and F) Reproduced with permission: 2019, American Chemical Society^[88]^

Optothermal microbubbles are generated due to the laser heating of light-absorbing substrates or light-absorbing particles. In some instances, microbubbles are also formed due to two-photon/multi-photon absorption-mediated heating of liquid at the substrate-liquid interface. We first consider bubble nucleation on light-absorbing plasmonic substrates. For light-absorbing plasmonic substrates such as gold nanoislands, discrete nucleation sites are densely located on the substrate to facilitate bubble formation during the printing. Figure 3A shows the temperature distribution around a laser-generated microbubble using computational fluid dynamics simulations. The temperature of the plasmonic substrates can reach beyond 120°C prior to bubble nucleation,^[49]^ whereas, for the light-absorbing metal particles, the temperature of particle surfaces can reach beyond 700°C.^[85]^ The vapor close to the thermal hotspot is superheated, and the temperature within the bubble decreases with increasing distance from the hot surface. The resultant temperature gradient causes a surface tension gradient along the bubble interface, which results in Marangoni convection with a spatially varying velocity distribution, as shown in Figure 3B. Rajeeva et al. determined the trajectories of metal ions in the solution under the influence of plasmonic bubbles using a combination of computational fluid dynamics and random walk simulations (Figure 3C).^[53]^ The results revealed that only a small fraction (~5%) of metal ions in the solution reached the three-phase contact line, which could be used for predetermination of the precursor concentration for a required linewidth of a printed structure.

“What forces print the particle on the substrate?” is one of the fundamental questions in BPL. Research has revealed that a combination of several distinct forces act on a particle as shown in Figure 3D. First, a particle away from the microbubble is dragged toward the three-phase contact line due to the Marangoni convection. When approaching the microbubble, the particle is then dragged toward the three-phase contact line by an evaporative force induced by continuous evaporation of the superheated liquid between the bubble and the substrate (middle panel in Figure 3D).^[86]^ As the particle touches the bubble surface, evaporative force, capillary force (from bubble surface distortion), electrostatic force, and Van der Waals force act on the particle.^[87]^ Since the bubble surface is almost a rigid interface due to the pressure difference between inside and outside the bubble, the downward capillary force on the particle is extremely high, which can overcome the electrostatic and Van der Waals forces. The net printing force enables the immobilization of particle on the substrate. It should be noted that the direction of the capillary force is strongly dependent on the contact angle of the bubble. Thus, there are variations in the printing capability for different solute-solvent-substrate systems.

In the case of light-absorbing particles as the heating sources, the force analysis for particle immobilization remains the same, while the mechanism of particle trapping and accumulation is different. For well-dispersed light-absorbing particle dispersion, the bubble nucleation sites are the particles themselves, which are mobile and homogenously distributed throughout the volume above the substrate. Zhang et al. performed a detailed analysis of bubble generation due to volumetric heating of the dispersion and the resultant accumulation of particles due to both stationary and moving bubbles.^[88]^ Briefly, the metal particles are excited with a resonant femtosecond laser beam to generate and grow the bubbles to a certain size.^[89]^ Because of the existence of metal nanoparticles in the region above the bubble surface, the transmitted laser beam excites the metal particles, and the temperature increases on the top of the bubble (Figure 3E). The surface tension gradient is pointed downward along the bubble interface, which slides the particles toward the substrate and immobilizes them on the substrate (Figure 3F). As the laser beam moves for continuous printing, the laser beam is refracted at the bubble interface, which creates a temperature hotspot in the direction of laser movement and causes an additional drag force on the particles. The accumulated particles on the substrate near the three-phase contact line act as local heat sources and cause the bubble to move forward. The significant difference between light-absorbing substrates and light-absorbing particles is the effect of bubble motion. The bubble motion in the former case causes particle immobilization at a new position,^[49]^ while in the latter case, particle immobilization happens at the existing position to move the bubble to a new equilibrium position due to asymmetric heating.^[88]^

## 3 | APPLICATIONS OF BPL

The accumulation and patterning of particles, ions, and molecules are essential in many emerging applications.^[90–92]^ Diverse nanoparticles and molecules patterned in selected configurations have been implemented for electronic, biological, and chemical applications.^[93–96]^ Accumulated nanoparticles have also been implemented in optical metamaterials and chiral metamolecules.^[100,97–99]^ Compared to other lithography techniques, BPL has a unique advantage in concentrating particles locally before their immobilization on the substrates. This concentrating capability enables the use of diluted inks with a low amount of materials for less wastage and easier processing rather than commercial viscous nanoparticle inks. Moreover, the localized high temperature and pressure at the three-phase contact line can be exploited as a local chemical nanoreactor to intensify chemical reactions and drive unconventional reaction pathways, which facilitate the in situ synthesis of complex inks and structured novel nanomaterials. In this section, we highlight the prominent applications of BPL, including quantum dot (QD) display, clinical diabetes detection, temperature-sensitive protein sensing, gas sensing, and microrobotics.

### 3.1 | BPL for QD patterning

**FIGURE 4:**
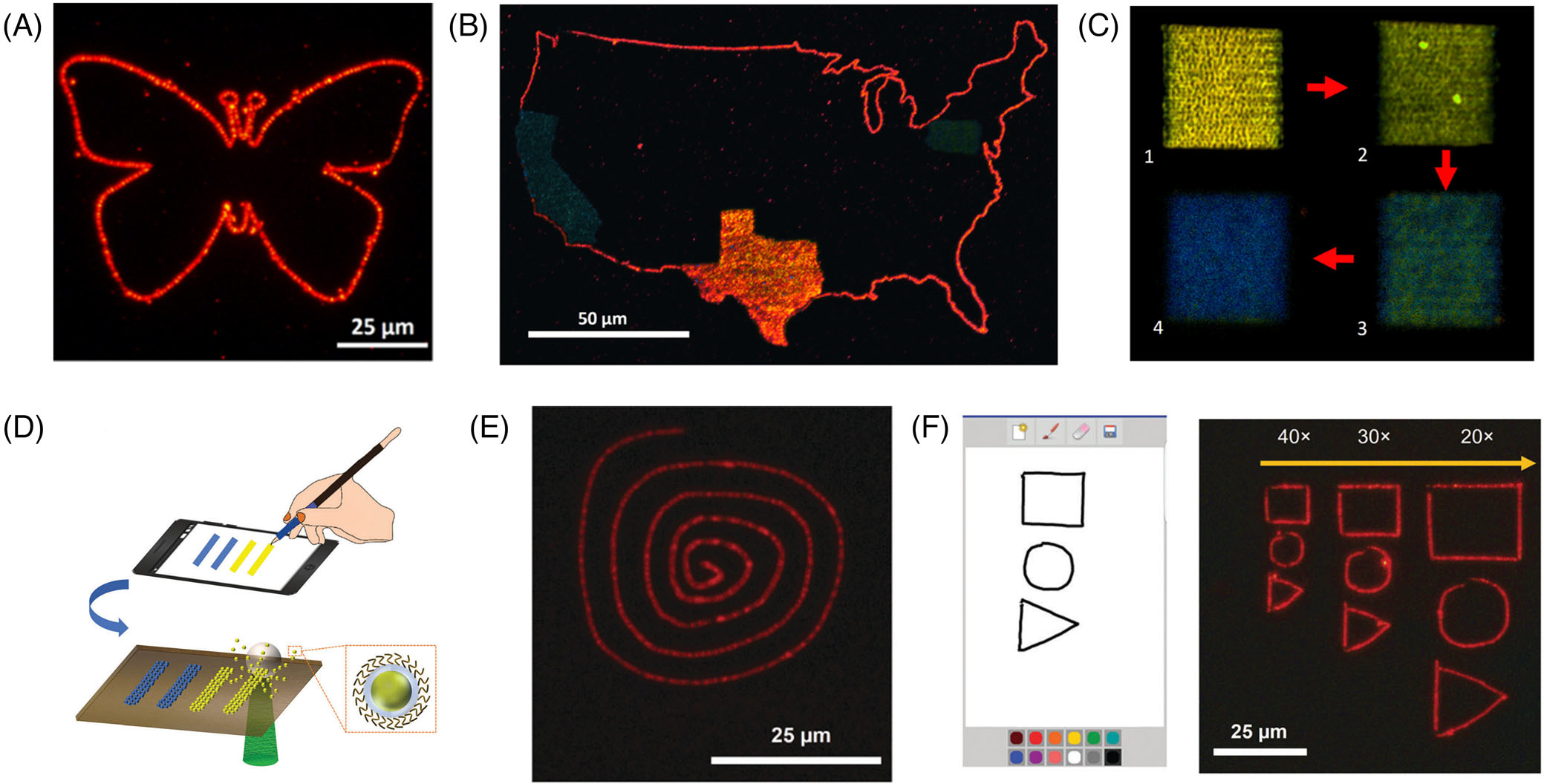
Bubble-pen lithography (BPL) for quantum dot (QD) patterning. (A) Fluorescence image of a butterfly pattern composed of red QDs. (B) Multistep BPL leading to a multicolor US map with the states of Texas, California, and Pennsylvania printed with different QDs. (C) Merged fluorescence images of yellow QDs printed in 20 *μ*m × 20 *μ*m squares with different parameters. Optical power, stage-translation speed, waiting time between neighboring lines, and line spacing for squares 1–4: (1) 0.52 mW/*μ*m^2^, 1000 *μ*m/s, 500 ms, and 1 *μ*m; (2) 0.54 mW/*μ*m^2^, 500 *μ*m/s, 600 ms, and 1 *μ*m; (3) 0.56 mW/*μ*m^2^, 100 *μ*m/s, 800 ms, and 1 *μ*m; and (4) 0.58 mW/*μ*m^2^, 100 *μ*m/s, 1 s, and 0.5 *μ*m. (A–C) Reproduced with permission: 2017, American Chemical Society.^[56]^ (D) Illustration of smartphone-controlled BPL. QDs are immobilized on a substrate to create patterns with different emission wavelengths based on the variable hand movement on the phone screen. (E) QD printing of a hand-drawn spiral pattern on a smartphone screen. (F) QD printing of hand-drawn patterns combined with scaling factors. (D–F) Reproduced with permission: 2017, The Royal Society of Chemistry^[79]^

QDs are known for their tunable optical properties with narrow emission bandwidth and high quantum efficiency.^[101,102]^ They have been used in many applications, such as displays,^[103]^ nanolasers,^[104]^ photodetectors,^[105]^ and electrocatalysis.^[106]^ The structured patterning of QDs on solid substrates is essential for many of these applications. Currently, the widely used inkjet printing technique for QD patterning suffers from several limitations such as nonuniform luminescence due to the coffee ring effect, poor printing resolution leading to large display pixels, and long postprocessing time required for the solvent evaporation from the patterned QD droplets. BPL is capable of overcoming these limitations. As an initial demonstration, Rajeeva et al. patterned a butterfly using red QDs without spreading or distortion of the printed lines (Figure 4A).^[56]^ They also achieved multicolor QD printing, where QDs of different emission wavelengths were utilized to pattern the United States map (Figure 4B). The authors further demonstrated the printing of QDs on flexible PET substrate using BPL^[56]^ for such applications as flexible and wearable displays.^[107]^

BPL has also been exploited to modify the fluorescence characteristics of QDs. Tuning the laser power and exposure time was used to control the photon-induced oxidation of QDs^[108]^ during the patterning process. The formation of the oxide layer on the QDs during BPL reduces the effective diameter of the QDs, which causes an increase in the quantum confinement and bandgap energy. This controllability is revealed by the tunable emission of the patterned QDs (Figure 4C).

To explore the versatility of BPL and enhance the user experiences, Zheng and coworkers developed smartphone-controlled BPL, which takes a manually drawn pattern on a smartphone as the input and prints the corresponding QD patterns onto the substrate (Figure 4D).^[79]^ Figure 4E shows the example of a printed QD spiral pattern that was drawn on the smartphone. Given the digital nature of this technique, the printed QD patterns can be easily altered or scaled on-demand via programmable control (Figure 4F). In addition, by tuning the handwriting speed on the smartphone, the printing speed of BPL can be altered to tune the fluorescence emission as a function of human physical motion. BPL with such versatile operation can serve as a highly accessible tool to manipulate matter at the nanoscale. With the further development along this line, one will expect smart BPL where internet-of-things, robotics, and cloud computing, and artificial intelligence are synergized to achieve digital micro/nanomanufacturing with full automation, high speed, mass customization, and global collaboration.

The implementation of BPL for QD patterning has immense potential in many applications because QDs are not only used for displays but also in a variety of sensors.^[109–111]^ Through the versatile control of BPL parameters, the patterned QDs can be tuned for optimal device performance. Furthermore, the plasmonic substrates used in BPL can also be utilized to enhance the performance of QD-based devices via plasmon-enhanced QD emission.^[112]^

### 3.2 | BPL for clinical detection of diabetes

**FIGURE 5:**
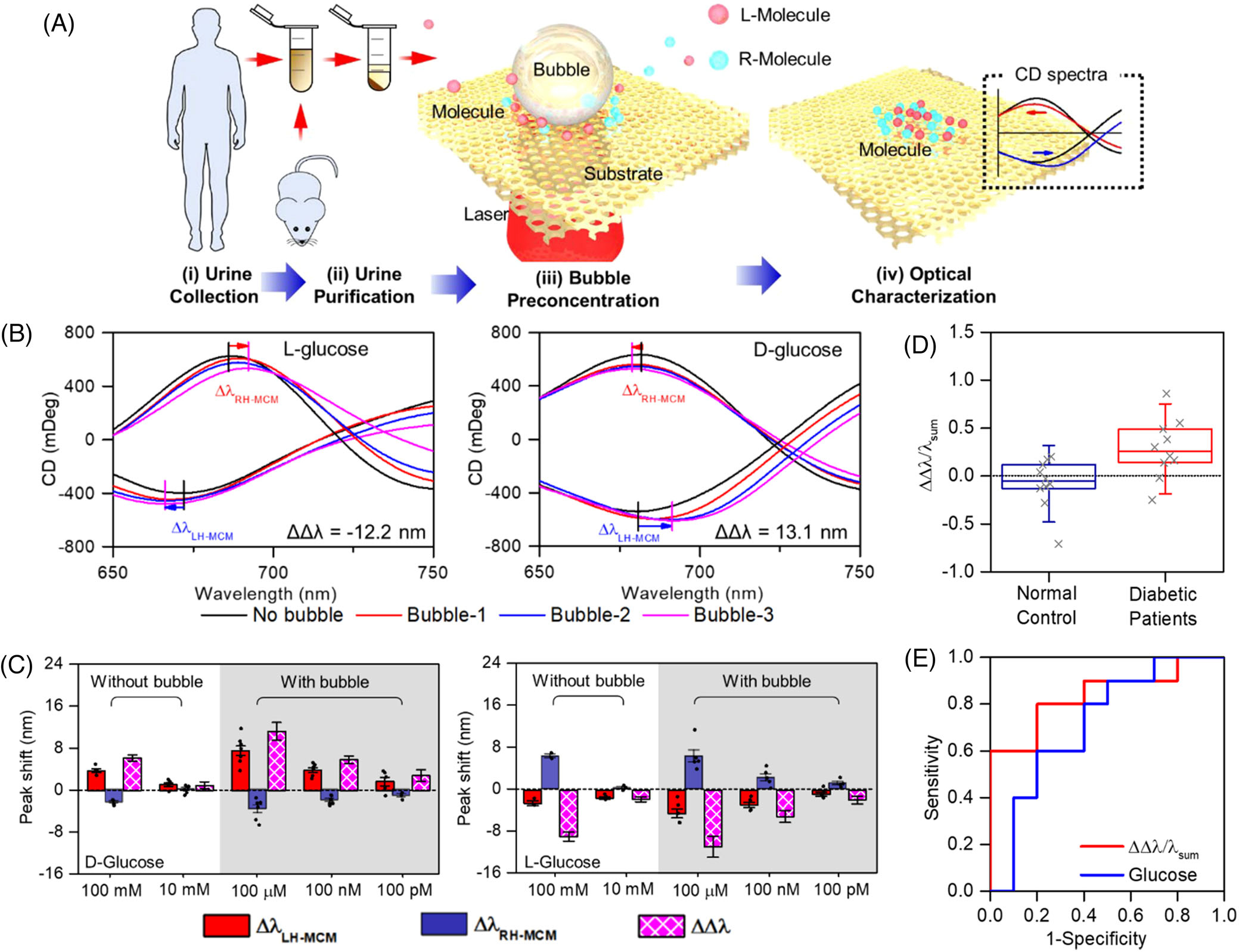
Bubble-pen lithography (BPL) for clinical detection of diabetes. (A) Schematic of the collection, purification, accumulation, and immobilization of metabolites in urine for clinical detection of diabetes based on the molecular-chirality-dependent circular dichroism (CD) spectral shifts. (B) Evolution of CD spectra of left-handed and right-handed moire chiral metamaterial (MCM) through the successive microbubble-assisted accumulation of L-glucose and D-glucose. (C) CD spectral shifts (Δ*λ*) and dissymmetry factors (ΔΔ*λ*) due to adsorption of D-glucose and L-glucose with and without the use of the microbubble to accumulate the molecules. BPL enhanced the detection limit by eight-fold. (D) Normalized dissymmetry factors (ΔΔ*λ*/*λ*_sum_) for urine samples from normal and diabetic humans. (E) Receiver operating curves of normalized dissymmetry factor and glucose concentration to measure the accuracy of the testing process with the bubble concentration time of 5 s. (A–E) Reproduced with permission: 2021, American Chemical Society^[113]^

The capability of concentrating and printing molecules makes BPL a desirable tool that can be integrated into various sensors for the detection of low-concentrated biomolecules for disease diagnosis. Liu et al. implemented BPL on plasmonic chiral metamaterials for the diagnosis of diabetes through the ultrasensitive label-free detection of abnormal chiral metabolites in urine.^[113]^ First, the urine samples were purified through centrifugation to remove all macromolecules and cells (Figure 5A). A plasmonic moire chiral metamaterial (MCM)^[114–116,51]^ was used to generate optothermal microbubbles and enable the high-sensitive chiral measurement of immobilized molecules with plasmon-enhanced superchiral fields. The total molecular chirality was measured by recording the circular dichroism (CD) spectra.^[117]^ Bubble-induced accumulation and printing of molecules on the plasmonic substrate causes different shifts in the CD peaks or dips of right-handed and left-handed MCMs (Figure 5B). By measuring the CD spectral shifts and the corresponding dissymmetry factors (the difference between the CD spectral shifts of left-handed and right-handed MCMs), abnormalities in molecular chirality of the lower-concentrated metabolites associated with diabetes are revealed. Figure 5C shows the measured dissymmetry factors due to the accumulation and printing of the molecules. The dissymmetry factor for BPL-assisted accumulation at the lower concentrations (100 pM–100 *μ*M) of molecules is comparable to the dissymmetry factors for the non-BPL-assisted accumulation at a much higher concentration (10–100 mM). This result shows that the detection limit for BPL-assisted accumulation is enhanced by seven orders of magnitude, which is promising for the earlier disease diagnosis. By comparing the normalized dissymmetric factors for normal and diabetic urine samples, one can see that the diabetes-positive clinical sample has a larger normalized dissymmetry factor (Figure 5D). The accuracy of detection is validated with receiver operating curve analysis (Figure 5E), confirming a higher diagnostic accuracy of 84% than that of conventional enzyme tests at ~72%.

Many chiral molecules in human body exhibit the phenomenon of homochirality, where one of the enantiomers is dominant in concentration than the other.^[118,119]^ Progress in life sciences has revealed that more diseases can affect the relative concentrations of these enantiomers, which can be exploited for disease detection.^[120]^ Compared with other chiral sensing techniques,^[121]^ bubble accumulation-assisted sensors have the unique advantages of (1) ultrahigh sensitivity (~100 pM concentration), (2) low cost, and (3) high detection efficiency without sophisticated derivation and labeling, which are required with other methods, such as liquid chromatography coupled with mass spectrometry.

### 3.3 | BPL for enhanced analyte sensing in liquids

Recent years have witnessed rapid progress in exploiting BPL to enhance the sensing of various analytes in liquid environments. For instance, Kotnala et al. demonstrated microbubble-assisted nanoaperture-based particle detection with fluorescence microscopy.^[122]^ The bubble-induced analyte accumulation at the three-phase contact line and the plasmonic enhancement of optical signals from the accumulated analytes at the nanoapertures can overcome the diffusion-limited analyte delivery and increase the analyte fluorescence efficiency. As a result, the detection time was reduced from several minutes to less than 1 min, and the detection sensitivity was increased by one order of magnitude.

Although BPL can be readily implemented for the accumulation of a variety of samples on the substrates, the temperature increase at the three-phase contact could deteriorate the analytes. Two strategies have been proposed to overcome the high-temperature issue. Tokonami et al. deposited a 50-nm gold (Au) layer to absorb the laser beam and generate a microbubble.^[123]^ The continuous metal layer could effectively dissipate heat to reduce the effect of high temperature on the immobilized bacteria. A high concentration (10^7^ cm^−2^) of bacteria assembly with a high survival rate of 80–90% was demonstrated. Although the temperature is reduced by a certain amount, it might not be sufficient to pattern more fragile biomolecules or cells without destroying their viability. For instance, using microbubbles for the detection of protein molecules would denature the proteins, as the denaturation temperature ranges between 29 and 99°C.^[124]^

**FIGURE 6:**
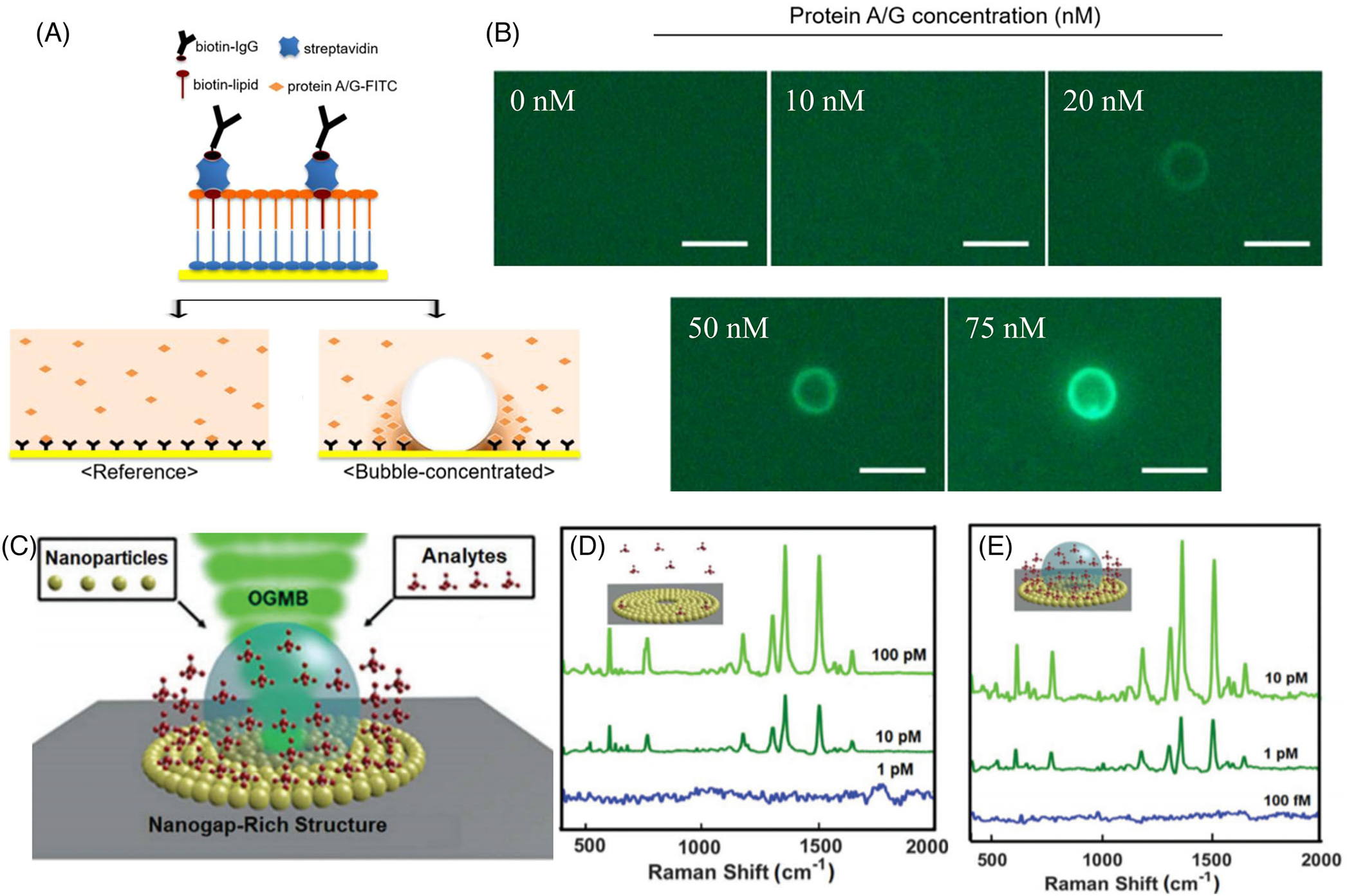
Bubble-pen lithography (BPL) for enhanced analyte sensing in liquids. (A) Schematic showing the immobilization and capture of protein with and without the use of microbubble in a bi-phasic system. (B) Fluorescence images after concentration of fluorescein isothiocyanate (FITC)-protein A/G with the use of microbubble at varying concentrations. Scale bar: 5 *μ*m. (A and B) Reproduced with permission: 2020, American Chemical Society.^[125]^ (C) Schematic of active sensing of analytes using BPL with nanogap-rich architectures. (D) Surface-enhanced Raman spectra of R6G analyte without bubble. (E) Surface-enhanced Raman spectra of R6G analyte assisted by BPL. (C–E) Reproduced with permission: 2019, The Royal Society of Chemistry^[127]^

To considerably reduce the temperature near the three-phase contact line and enable protein-protein interaction studies, Kim et al. proposed a biphasic system with perfluoropentane (PFP) droplets dispersed in water.^[125]^ PFP has a boiling point of 30°C, which makes it an ideal candidate for low-temperature BPL of proteins without denaturation. In addition, to enable the study of protein-protein interactions, the plasmonic substrate was modified with zwitterions to prevent unwanted immobilization of proteins during the BPL (Figure 6A). Fluorescence-labeled protein was used as a target sample to visualize the BPL-assisted surface capture event. Figure 6B shows the conjugated events in the event of microbubble accumulation for 1 min at different concentrations of fluorescent protein (0, 10, 20, 50, and 75 nM). This proof-of-concept work demonstrated the use of BPL to capture targeted proteins on functionalized substrates at low temperatures without any damage, which is essential for the broader implementation of BPL in biology. Biocompatible deposition of biomolecule-functionalized nanoparticles to optically transparent surfaces was demonstrated using shrinking surface plasmonic bubbles.^[126]^

Another approach to implementing BPL in analyte sensing is based on surface-enhanced Raman spectroscopy (SERS). Researchers have implemented BPL to pattern silver-ring-based Raman substrates for the optical detection of analytes at sub-micro molar concentrations.^[52]^ Upon the optothermal generation of bubbles, silver ions of the precursor solution were reduced at the three-phase contact lines to produce the silver rings due to the high ion accumulation and temperature. Karim et al. applied BPL to pattern plasmonic nanoparticles into nanogap-rich structures on the substrates and then to concentrate analytes on the nanogap-rich structures for SERS (Figure 6C).^[127]^ Using Rhodamine 6G molecules as analytes, the authors demonstrated the bubble-enhanced SERS intensity for detecting low-concentrated molecules. The SERS-detectable molecular concentration is 10 pM without bubble (Figure 6D) and 1 pM with bubble (Figure 6E). Although this work involved BPL of nanoparticles and analytes in sequential patterning steps, the nanoparticles could also be mixed with analytes for single-step patterning.

### 3.4 | BPL for gas sensing

**FIGURE 7:**
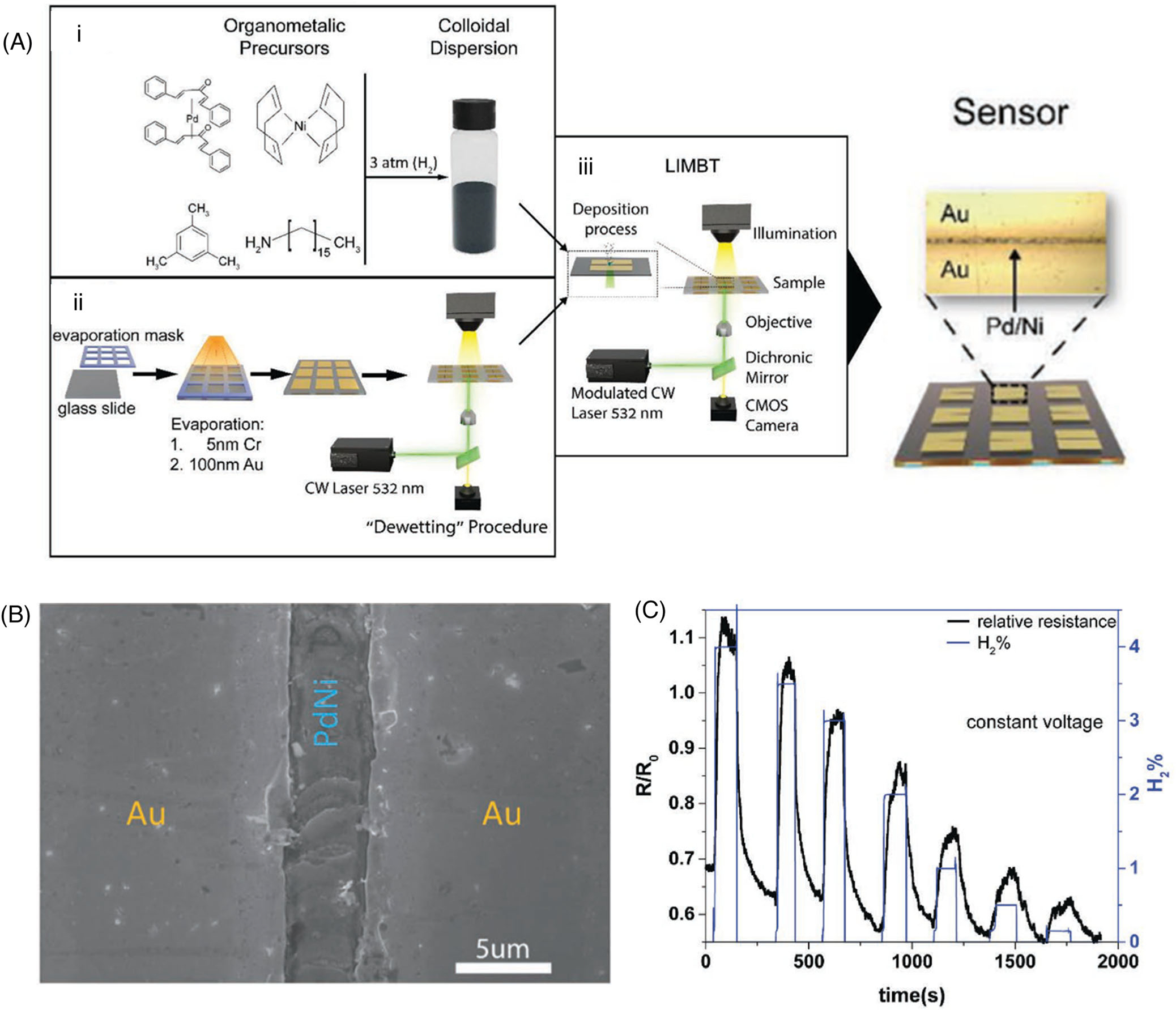
Bubble-pen lithography (BPL) for gas sensing. (A) Flowchart outlining the fabrication of the sensor: (i) synthesis of organometallic-based nanoparticles; (ii) construction of conductive pads; (iii) deposition of the nanoparticles using BPL. (B) Scanning electron micrograph of the PdNi nanoparticle deposition between two Au electrodes. (C) The sensor response to various H_2_ concentrations with a constant voltage. (A–C) Reproduced with permission: 2019, WILEY-VCH^[133]^

Hydrogen presents as an attractive, clean energy alternative to replace fossil fuels due to its high energy density, and cost reduction of its production enables the realization of energy production in diverse applications at all scales.^[128–130]^ Since hydrogen is a colorless, odorless, and highly flammable gas, the detection of production and leakage of hydrogen is essential to facilitate its applications as a safe energy source.^[131,132]^ Palladium (Pd) is widely used to detect hydrogen via dissociative absorption. Current fabrication methods for Pd-based hydrogen sensors involve a two-step process: synthesis of Pd crystals and deposition of the crystals into prescribed patterns. BPL can achieve single-step fabrication of Pd sensors from the palladium precursor solution (Figure 7A).^[133]^ A hydrogen gas sensor was prepared by printing colloidal Pd/Ni alloy nanoparticles in a predetermined path between Au pads (Figure 7B). As hydrogen gas was absorbed, the printed palladium expanded and created new pathways for electrons to flow, decreasing the resistivity between the Au pads. The resultant hydrogen sensor had a detection limit of 100 ppm H_2_ in air at room temperature (Figure 7C).^[133]^

### 3.5 | BPL for catalysis

**FIGURE 8:**
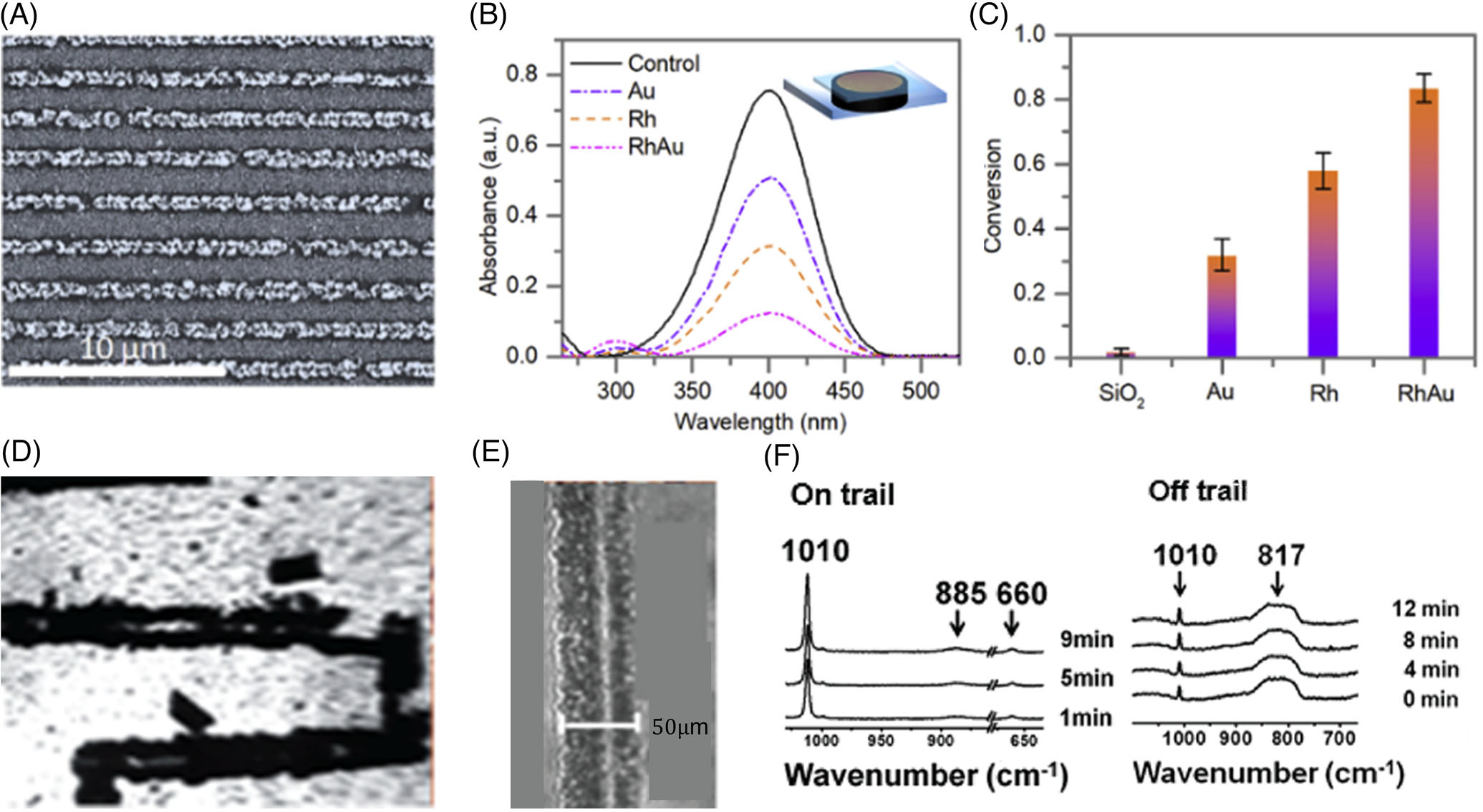
Bubble-pen lithography (BPL) for catalysis. (A) Scanning electron micrograph of bubble-printed nanoalloys composed of gold and rhodium with a 2-*μ*m line spacing. (B) UV-vis absorption spectra demonstrate the reduction of *p*-nitrophenol by the printed Au-Rh nanoalloys as catalysts. (C) Conversion percentage of reactants into products shows that printed Au-Rh lines are more efficient for catalytic applications compared to SiO_2_, Au, and Rh control samples. (A–C) Reproduced with permission: 2019, Elsevier Inc.^[53]^ (D) Optical image of bubble-printed soft oxometalate–porous organic framework (SOM-POF) composites for catalytic oxidation of benzaldehyde into benzoic acid. (E) The resolution of BPL of SOM-POF trails. (F) Raman spectroscopy depicting catalysis at trail site (on trail) versus away from the trial site (off trail). An increase in the intensity of the product benzoic acid (1010 nm peak) indicates the site-specific nature of catalysis. (D–F) Reproduced with permission: The Royal Society of Chemistry^[137]^

Metal nanoalloys have demonstrated enhanced catalytic properties compared with their individual counterparts.^[134]^ However, synthesis of such nanoalloys, is difficult due to the complexity of synthetic techniques and chemical precursors.^[135]^ This complexity arises from the preparation and containment of reagents in nanoscale spaces such as micelles.^[136]^ Laser-induced microbubbles create an environment where high temperature and high precursor accumulation lead to the fast and micelle-free synthesis of nanoalloys.^[133]^ To explore the formation dynamics of nanoalloys and their enhanced catalytic property, Zheng and coworkers have achieved a microbubble-mediated accumulation of Au and rhodium (Rh) ions for Au/Rh nanoalloy formation. Figure 8A shows the bubble printing of microscale Au/Rh nanoalloy lines from their precursor mixtures. To test the catalytic performance of the alloys, the authors carried out a reduction reaction of *p*-nitrophenol in the presence of pure Au, pure Rh, and the alloys. Figure 8B shows the ultraviolet (UV)-visible (vis) absorption spectra revealing the reduction reaction catalyzed by the different catalysts. A higher conversion percentage of the reactants catalyzed by the alloys demonstrates a better catalytic performance of the alloys than pure metals (Figure 8C).

In another example, BPL was implemented to fabricate a catalytic chip based on a soft oxometalate (SOM) and a porous organic framework (POF) material.^[137]^ Figure 8D shows the printed architecture of the SOM-POF composite. The chip was designed with various SOM-POF composites and tested against benchmark molecular catalysts, displaying high catalytic activity due to the high accessibility of mesospheres. The BPL-based trail had a resolution of 50 *μ*m (Figure 8E). Figure 8F quantifies the catalytic ability through Raman peaks corresponding to the oxidation of benzaldehyde to benzoic acid. The peaks corresponding to the reaction products increase with time only on the printed trail, demonstrating the site-specific nature of the catalysis.

In conclusion, the high-temperature zone at an optothermally generated bubble with highly concentrated ions creates a favorable environment for intensified nanoscale alloying.^[138–141]^ With the capability of in situ synthesis and structuring of nanoalloys, BPL is instrumental in creating architected nanoalloys that exhibit superior catalytic activities without postprocessing.

### 3.6 | BPL for microrobotics

**FIGURE 9:**
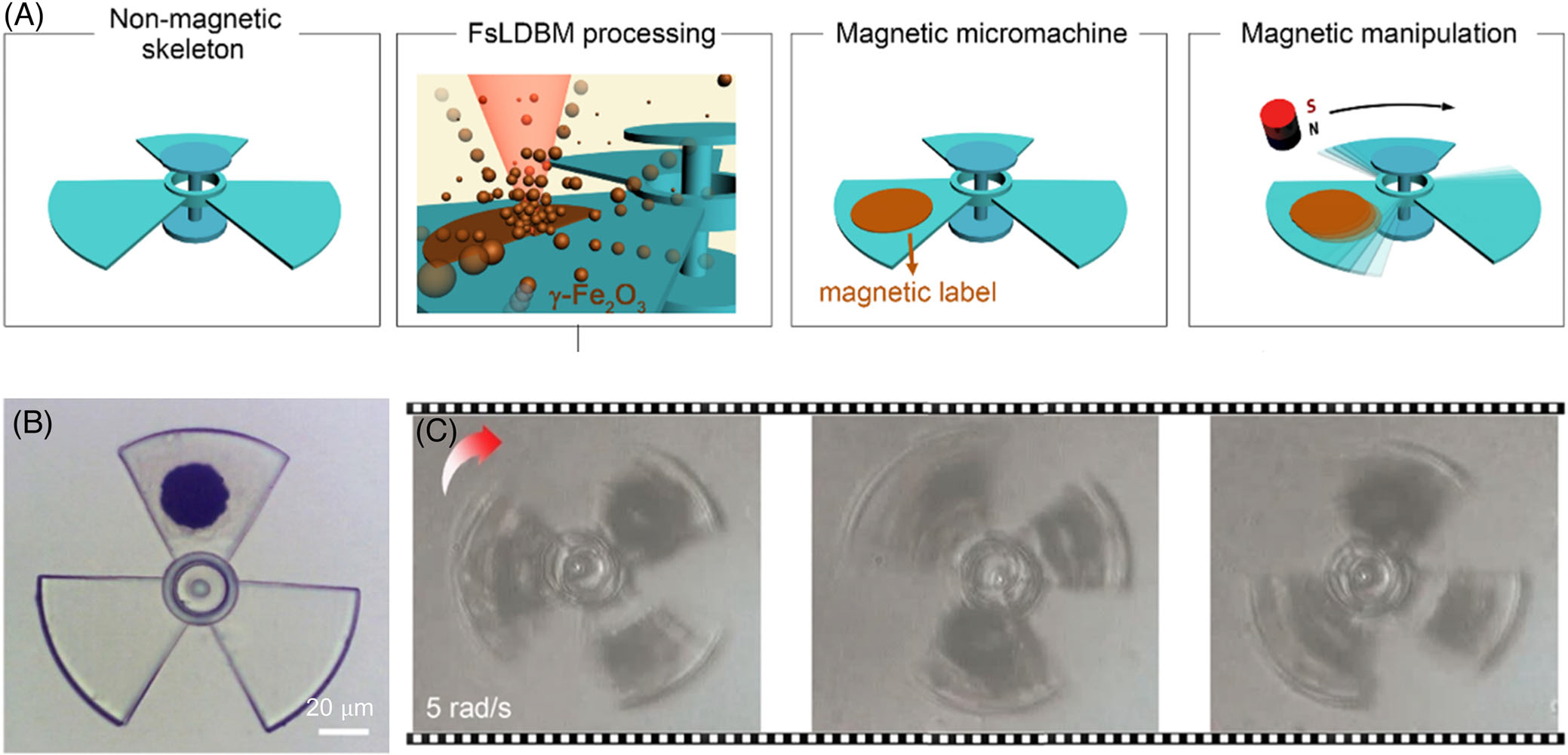
Bubble-pen lithography (BPL) for micromachines. (A) Schematic outlining the micromachine skeleton and the printing of *γ*-Fe_2_O_3_ nanoparticles on a micromachine via BPL using a femtosecond laser. The printed magnetic nanoparticles act as a magnetic label, enabling the motion of the micromachine under an external magnetic field. (B) Optical image of a micromachine after BPL of nanomagnets. (C) Time-lapse images of micromachine rotation demonstrate the conversion of a passive structure into an active machine. (A–C) Reproduced with permission: 2021, American Chemical Society^[87]^

Manipulation of micro-objects and biological materials is instrumental in diverse applications such as drug delivery, cell-cell interactions, and colloidal metamaterials.^[142–145,70]^ Many micro-/nano-manipulation techniques exploiting electrical, thermal, optical, magnetic, and acoustic fields have been developed.^[146–152]^ Magnetic manipulation is well-known for its noninvasive nature, high penetration depth, and high biocompatibility.^[153,154]^ However, magnetic manipulation is limited to magnetic materials. Wang et al. overcame this limitation by implementing BPL for the fabrication of magnetic nanoparticles on various microscale and milliscale objects as substrates.^[87]^ Specifically, they used a femtosecond laser for microbubble formation at the interface of the substrate and magnetic nanoparticle solution through two-photon absorption. Compared to the plasmonic-heating-mediated bubble generation, two-photon absorption requires higher laser power. The two-photon method is effective for BPL because the generated bubble can still drive Marangoni convection that attracts colloidal particles in the solution toward the substrate (Figure 9A). For instance, a 20-*μ*m circular area of magnetic particles was deposited on a 100-*μ*m nonmagnetic microstructure (Figure 9B). When exposed to an external magnetic field (e.g., a magnet), the magnetic nanoparticle aggregate on the microstructure responded to the external magnetic field, driving the microstructure to rotate at a high speed of ~1900 revolutions per second (Figure 9C). To demonstrate the wide applicability of BPL, printing of magnetic particles on different substrates such as glass, polymer, and biological material (living daphnia) was implemented. The on-demand BPL of magnetic particles and other functional materials on diverse substrates facilitate the fabrication of a wide range of hybrid structures for robotic and biological applications.

## 4 | PERSPECTIVE

BPL has demonstrated its viability in many distinct fields. BPL is compatible with a wide range of substrates, including transparent substrates (glass), biological substrates (living daphnia), and flexible substrates (PET). In addition, the high integrability of the remotely controlled BPL with microfluidic systems is paving a way toward continuous cycling of multiple precursor solutions for automatic multi-material printing. Table 1 summarizes the recent progress of BPL in terms of applicable substrate, printed material, printing mode, printing resolution, laser wavelength and power, and printing speed. Despite the many advantages of BPL, one needs to address several issues for BPL to be implemented for a broader range of applications.

### 4.1 | Thickness and morphology control

Optical heating strongly depends on the light-absorbing materials in the BPL system, which can be substrates or nanoparticle dispersions. In both scenarios, the deposited materials on the substrates can alter the local absorption of the laser beam and distort the bubble size during the patterning process.^[155]^ The effect of the deposited materials becomes more prominent while performing multi-pass BPL for greater thickness. Although the thickness of the patterned structures increases during multi-pass, the rate at which thickness increases might not be a constant due to the influence of the deposited materials. A comprehensive relationship among material delivery, concentration, immobilization, and other physiochemical processes must be established for any targeted BPL system to precisely control the thickness of printed patterns.

Similarly, the morphology of the printed patterns is currently not well controlled. Understanding the aggregate formation or chemical reaction near the three-phase contact line is essential to the printing of ink particles or reaction products into patterned structures of desired morphology. While smooth patterns are usually favorable for electronics, other applications such as catalysis benefit from the high surface roughness of the printed structures. Thus, one should achieve on-demand control of the morphology of patterned structures to enable the widespread applicability of BPL to different applications.

### 4.2 | Bubble stability and motion stability

Two questions remain unanswered for high-speed laser scanning in the high-throughput BPL. One is whether the same bubble is stably manipulated for uniform patterning. The other is whether the Marangoni convection turns turbulent. For continuous patterning using BPL, the microbubble must exist throughout the patterning time. In discrete patterning, the microbubble is directed away from the substrate due to the sudden absence of laser heating and high buoyancy force on the bubble. However, new bubbles are formed immediately (in several milliseconds) at the new laser spots. This cyclic process of bubble generation, bubble movement, and bubble emigration from the substrate must be well understood and controlled to achieve any desired patterning. For example, small deficits in the patterns could occur during the bubble emigration stage, which result in electrical breakage of the printed conductors. To overcome this limitation, a better adhesion between the bubble and the substrate is necessary. In order to push toward the high-throughput BPL with high-speed laser scanning, one should employ an iterative process of substrate functionality design and study of the adhesion and drag forces on the bubble to develop a better understanding and control of bubble dynamics. A quantitative description of the bubble stability in terms of average bubble generation time and average bubble lifetime is required for better comparison across future works. An alternative method of understanding the efficiency of BPL as a lithographic approach is to quantify printing defects for benchmarking this technology for practical applications.

During the extremely fast BPL, the high-speed bubble motion might result in a large Reynolds number, pushing Marangoni convection into the turbulent regime. Thus, another future research direction in high-throughput BPL is to understand the transition of convection into turbulent flows at high laser speeds and its impacts on patterning and alloying at the nanoscale. Alternatively, to overcome the throughput limitation in BPL due to a lack of understanding and control of bubble dynamics at high speeds, digital micromirror devices and spatial light modulators can be used to split one laser beam into multiple laser beams with independent control to opothermally generate multiple bubbles for parallel BPL. This multiple-beam flexibility can significantly increase the throughput of BPL, featuring advantages over other printing techniques like inkjet printing with a limited number of nozzle heads. However, a full understanding of interactions among different flows arising from individual bubbles in the arrays is needed to ensure the high-throughput BPL of desired structures in parallel.

## 5 | CONCLUSION

We have reviewed progress in both experimental and theoretical fronts of BPL. Compared to other lithographical approaches, BPL has the unique advantages of concentrating ions, molecules, nanoparticles, and biomaterials before patterning them on the substrates. Such an accumulation process enables the use of low-concentrated solution-based inks or analyte samples without extensive pre-processing. Moreover, the small ink or sample volume (~100 *μ*L) along with the low material wastage enables BPL to be a table-top prototyping apparatus or a point-of-care device. With the continuous efforts in advancing fundamental understanding of light-matter interactions and opto-thermo-fluidic multiphysics, colloidal chemistry, and instrumentation from multidisciplinary communities, we envision that BPL will be further improved to positively impact diverse industrial domains such as energy, information technology, and health care.

## Figures and Tables

**TABLE 1:** A summary of recent progress of bubble-pen lithography (BPL)

Substrate	Material	Printing mode	Laser wavelength	Laser power/intensity	Feature size	Printing speed	Reference
Au nanoislands on glass or PET	PS microparticles	Discrete	532 nm	0.56 mW/*μ*m^2^ (0.43 mW)	~2 *μ*m	–	[49]
	Quantum dots	Continuous	532 nm	0.3 mW/*μ*m^2^ (0.23 mW)	500 nm	10 mm/s	[56, 79]
	Au and Rh precursors	Continuous	532 nm	0.6 mW/*μ*m^2^ (0.47 mW)	550 nm	40 *μ*m/s	[53]
	Silver precursor	Discrete	532 nm	0.2 mW/*μ*m^2^ (0.16 mW)	<1 *μ*m (Ring: 3 *μ*m)	–	[52]
Au on Glass	Pd and Ni precursors	Continuous	532 nm	150 mW	4 *μ*m	30 *μ*m/s	[133]
Glass	Polyaniline nanoparticles	Continuous	532 nm	2.8 mW	2 *μ*m	0.8 mm/s	[57]
	Soft/poly oxometallates	Continuous	1064 nm	25 mW	5 *μ*m	–	[137]
	Soft/poly oxometallates	Continuous	1064 nm	35 mW	5 *μ*m	1 mm/s	[78]
		Discrete			3 *μ*m		
	Iron oxide/Silver precursors		532 nm	13 mW	(Ring: 10 *μ*m)	1 *μ*m/s	[156]
		Continuous			5 *μ*m		
Glass/Polymer/Living substrates	Magnetic microparticles	Continuous	800 nm (fs laser)	10 mW	1 *μ*m	–	[87]

Abbreviations: BPL, bubble-pen lithography; PET, polyethylene terephthalate; PS, polystyrene.

## Data Availability

None.
